# Cow milk is an important source of iodine for prenatal health, and switching to plant-based milk can lead to iodine insufficiencies

**DOI:** 10.3168/jdsc.2023-0424

**Published:** 2024-02-01

**Authors:** Hallie Lundquist, Julie Hess, Madeline Comeau, Joanne Slavin

**Affiliations:** 1 Department of Food Science and Nutrition, College of Food Agricultural and Natural Resource Sciences, University of Minnesota–Twin Cities, St. Paul, MN 55108; 2 United States Department of Agriculture, Grand Forks, ND 58203

## Abstract

Iodine insufficiencies are common among many populations, particularly pregnant women. One of the main functions of iodine is making thyroid hormone. The 2 main hormones that iodine influences are triiodothyronine and thyroxine. Thyroid hormone affects metabolism of most tissues. For the average adult, the recommended dietary allowance (RDA) for iodine is 150 µg. During certain stages of life, such as pregnancy, lactation, and infancy, the importance of iodine is even greater as it supports brain, bone, and organ development. The RDA for iodine during pregnancy is 220 µg and, during breastfeeding, the RDA is 290 µg. Consuming enough iodine in the diet during pregnancy helps support fetal neurodevelopment. Iodine is found in several food sources such as seafood and iodized salt; however, dairy products are one of the major sources of iodine in American diets. It is important to note that only bovine milk products are rich in this mineral. One cup of milk provides 39% and 57% of the daily iodine needs for the average adult woman and pregnant woman, respectively. As the Dietary Guidelines for Americans (DGA) recommend limiting sodium intake, which includes iodized salt, dairy may be an especially important source of iodine. However, according to the USDA, about 90% of the US population does not meet the dairy recommendations presented in the DGA. In recent years, plant-based diets have received a lot of attention. A market for plant-based milk alternatives has grown and includes a variety of options such as almond, soy, and oat milk. Plant-based milks do not naturally contain iodine and are typically not fortified with iodine. Women of childbearing age who drink plant-based milks instead of cow milk have lower urinary iodine concentrations than women who consume cow milk. This review will focus on the importance of iodine in the diet to support prenatal health, lactation, and infant health.

## Graphical Abstract

**Figure:**
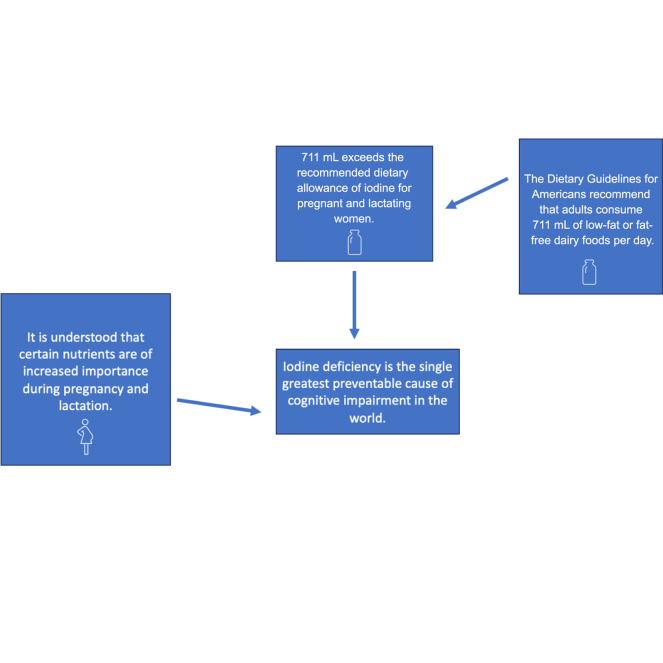

Iodine is an important trace mineral necessary during all stages of life. It cannot be synthesized in the body, so iodine must be consumed in the diet. Iodine is important for development and growth and aids in the body's ability to use nutrients. Iodine acts as a cofactor for thyroid hormones triiodothyronine (**T3**) and thyroxine (**T4**; Lee et al., 2016). Working together, T3 and T4 aid in metabolism and growth for the body's cells. Thyroid-stimulating hormone (**TSH**) is responsible for much of the thyroid function as it allows for the intake of iodine by the thyroid gland (Shahid et al., 2023). As part of the endocrine system, the hypothalamus secretes thyrotropin-releasing hormone (**TRH**), which then signals the pituitary gland to secrete TSH (Institute for Quality and Efficiency in Health Care, 2021). Thyroid-stimulating hormone will then signal the thyroid hormone (**TH**) to secrete T3 and T4 hormones into the bloodstream (Institute for Quality and Efficiency in Health Care, 2021). The pituitary gland is responsible for determining how much T3 and T4 is secreted based on physiological needs (Institute for Quality and Efficiency in Health Care, 2021). Concentrations of T3 and T4 are regulated via a negative feedback loop. When levels of T3 and T4 decrease below normal levels, the pituitary gland is stimulated to produce TSH. Once the thyroid then increases blood concentrations levels of T3 and T4 back to their normal levels, the secretion of TRH by the hypothalamus and, in turn, the secretion of TSH by the pituitary gland, are inhibited.

The main functions of TH are to regulate metabolism and support growth and development. The secretion of TH is necessary from fetal development through adolescence to support myelination of the nervous system and cognitive development. Thyroid hormone continues to be important during adulthood to support healthy reproductive outcomes. Thyroid hormone is also important when the heart is beating faster and when body temperature is elevated (Institute for Quality and Efficiency in Health Care, 2021). When there is insufficient iodine in the body, TSH concentrations remain high, and the size of the thyroid gland increases with the decrease in TH production. This growth of the thyroid gland is given the clinical name of goiter. In cases where the thyroid cannot produce enough hormones for an extended period of time due to severe iodine deficiency, hypothyroidism can occur (Kapil, 2007). This condition prevents the body from proper metabolism, regulation of body temperature, and heart rate (Kapil, 2007). In severe cases of iodine deficiency during pregnancy, stillbirth and developmental issues can occur (Lee et al., 2016). In the maternal diet, iodine deficiency is the single greatest preventable cause of brain development issues in the world (de Benoist et al., 2008).

Severe iodine deficiency, which can lead to goiter formation, has been reported in the literature as occurring as far back as 2700 BC (Niazi et al., 2011). Much of what we know about goiter and the importance of iodine intake originates from research dating back to the 19th and 20th centuries (Niazi et al., 2011). In the 19th century, the scientist Eugen Baumann studied the thyroid gland of sheep and found a significant amount of iodine (Niazi et al., 2011). Later, Adolphe Chatin, a French chemist, proved that iodine derived from freshwater plants, such as seaweed and phytoplankton, could prevent goiter (Niazi et al., 2011).

Iodine is naturally present in the Earth's soil (Zimmermann, 2011). However, certain areas of the world that have experienced flooding, erosion, and other disruptions to the soil have less iodine in the soil, and therefore, less iodine in the foods grown in that soil. Historically, regions with high prevalence of goiter were sent salt from regions with no goiter prevalence. This exchange reduced the incidence of goiter. Although this proved the relationship between iodine and goiter, it took many years for commercially iodized salt to become readily available (Niazi et al., 2011). In the 20th century, David Marine proved the essentiality iodine for thyroid function (Niazi et al., 2011). In the 1920s, the Great Lakes, Northwest, and Appalachian regions were areas with the highest incidence of goiter and were coined the “goiter belt” (Leung et al., 2012). These regions lacked iodine-rich soil; therefore, food sources that grew in the soils had low iodine content (Leung et al., 2012). In 1922, at the Michigan State Medical Society thyroid symposium, David Cowie, the chairman of Pediatrics at the University of Michigan, proposed the addition of iodine in table salt because it is a cheap, accessible product used by everyone (Markel, 1987). Before the implementation of iodized salt, many places in the United States suffered from iodine deficiency (American Thyroid Association, 2023). Now, the incidence of goiter is limited in the United States; however, many places around the world still have high rates of iodine deficiency and goiter.

Iodine recommendations in the United States vary by age and life stage (Table 1). As iodine supports brain development, infants require greater amounts of iodine than older children. From early childhood through adulthood, iodine requirements increase. The life stages with the greatest iodine requirements are pregnancy and lactation. Due to iodine's critical role in thyroid function, sufficient iodine intake is crucial during pregnancy and lactation.

**Table 1:** Recommended dietary allowances (μg) for iodine (National Institutes of Health Office of Dietary Supplements, 2023)

Age	Male and female	Pregnancy	Lactation
Birth–6 mo	110	—	—
7–12 mo	130	—	—
1–8 yr	90	—	—
9–13 yr	120	—	—
≥14 yr	150	220	290

Throughout pregnancy, thyroid function is especially crucial. The iodine supply in the body must increase accordingly. Pregnancy is a notable time for not only the developing fetus, but also for maternal physiologic changes associated with gestation. These changes include increased TH production, increased renal iodine excretion, and physiologic accommodation for fetal iodine requirements (Alexander et al., 2017; National Institutes of Health Office of Dietary Supplements, 2023). These processes each require adequate maternal iodine status, and without adequate amounts, “the most damaging effect of iodine deficiency is on the developing brain” (Institute of Medicine, 2001, p. 261). To accommodate these demands of pregnancy and the developing fetus, the recommended dietary allowance (**RDA**) increases from 150 µg/d for nonpregnant adults to 220 µg/d for pregnant individuals (Institute of Medicine, 2001; National Institutes of Health Office of Dietary Supplements, 2023). The National Institutes of Health Office of Dietary Supplements lists pregnant individuals as a group at risk of iodine inadequacy due to increased needs (Institute of Medicine, 2001; National Institutes of Health Office of Dietary Supplements, 2023). National Health and Nutrition Examination Survey (**NHANES**) data from 2003 to 2014 indicate that a large portion of pregnant individuals in the US population did not fall into sufficient iodine ranges based upon spot urinary concentrations below the minimum adequacy threshold of 150 µg/L (National Institutes of Health Office of Dietary Supplements, 2023).

Some of the best natural sources of iodine are seafood products such as cod, seaweed, and oysters (National Institutes of Health Office of Dietary Supplements, 2023). However, for the general population, taste, and cultural preference affect how much seafood is consumed. Iodized salt is also an important source of iodine for Americans (US Food and Drug Administration, 2023). One gram of iodized salt contains 45 µg of iodine/gram of salt, so about three-quarters of a teaspoon (1,725 mg of sodium) fulfills iodine requirements for the average adult. Actions to combat deficits associated with iodine deficiency include the universal salt iodization recommendation of the World Health Organization (2022). The World Health Organization recommends using salt as a vehicle for iodine due to worldwide usage of salt in food (World Health Organization, 2022). Organizations, such as the American College of Obstetricians and Gynecologists, that provide health or dietary guidance for those who are pregnant recommend that women consume between 220 and 250 µg of dietary iodine per day, during pregnancy. Some of these organizations additionally recommend supplementation of at least 150 µg/d during pregnancy to ensure adequate intake (Rogan et al., 2014; Alexander et al., 2017; World Health Organization, 2022; National Institutes of Health Office of Dietary Supplements, 2023; American College of Obstetricians and Gynecologists, 2024).

Other important sources of iodine for Americans are dairy products. Milk, yogurt, and cheese are all good sources of iodine. For consumers who may have a lactose allergy, lactose-free milks also contain iodine. The season in which the milk is produced, pasteurization, farming practice (organic and nonorganic), and fat content can all contribute to differing concentrations of iodine in milk (O'Kane et al., 2018; Stevenson et al., 2018). In a study from the United Kingdom, researchers assessed the iodine content of organic milk and UHT processed milk during different seasons. Organic milk produced in the winter had 44% lower iodine concentrations than conventional milk (Stevenson et al., 2018). The UHT milk also had 27% less iodine than conventionally pasteurized milk. The reasons for differences in iodine concentration in cow milk are complex. Factors such as housing (i.e., year-round housing, summer-housing, winter-housing) as well as geographical location can affect iodine concentrations because iodine is atmospherically transferred into the soil and into the diet of cows through grazing (McKernan et al., 2020). Further research is necessary to determine how to keep iodine concentrations more consistent across these parameters.

However, the amount of iodine in cattle feed is the main contributor to the amount of iodine in milk (O'Kane et al., 2018). Iodine is incorporated in the diets of cattle as they also require iodine for thyroid gland functions (Schöne et al., 2017). Iodine requirements for dairy cattle are 0.5 to 1.5 mg/kg DM (Schöne et al., 2009). There is a direct linear relationship between iodine in cattle feed and milk iodine (Niero et al., 2023). Common feed types for farms are dependent on cattle breed as well as dairy farm practices. Iodine content in feed can vary (i.e., corn, grass, hay, *Ascophyllum nodosum* meal; Niero et al., 2023). The naturally occurring iodine in feed depends on the amount of iodine that is taken up from the soil by the plants (Niero et al., 2023).

In addition to iodine from the cow's diet, iodine can also enter milk products through use of iodized sanitizers in the milking process (Flachowsky et al., 2014). Some of the iodine from iodophor-based sanitizers used during the milking processes is transferred into the milk. The amount of iodine in milk that comes from sanitation practices may be negligible (Conrad and Hemken, 1978). One study compared iodine content in milk from cows treated with iodophor-based teat dips to milk from cows on which a tincture of iodine was sprayed on the skin between the vulva and udder (Conrad and Hemken, 1978). Iodine concentration was higher in the cows treated with iodine spray than from the cows treated with teat dips, indicating that iodine is absorbed into the skin of the cow and secreted into the milk (Conrad and Hemken, 1978). On average, 1 cup or 237 mL of nonfat milk contains 85 µg of iodine (Table 2; Institute of Medicine, 2001; Roseland et al., 2020; Ershow et al., 2022).

**Table 2:** Iodine content of dairy products and percent daily value (Dellavalle and Barbano, 1984; National Institutes of Health Office of Dietary Supplements, 2023)

Dairy product	Serving size	μg per serving	Percent daily value
Nonfat milk	1 cup or 8 oz (237 mL)	85	57
Nonfat Greek yogurt	3/4 cup (187 mL)	87	58
Chocolate ice cream	2/3 cup (158 mL)	28	19
Cheddar cheese	1 oz (28 g)	15	19
Whey protein powder	25 g	118	79

Bovine dairy products are good sources of iodine (Nutrition Source, 2023). The dietary guidelines for Americans (**DGA**) recommend that adults consume 3 cups of fat-free or low-fat dairy foods per day in the Healthy US-Style and Healthy Vegetarian Dietary Patterns (US Department of Agriculture and US Department of Health and Human Services, 2020; Stewart and Kuchler, 2022). This recommendation provides amounts of iodine that exceed the RDA for average adults and meets the requirements for pregnant and lactating women. Dairy foods can be an important source of iodine during pregnancy (Whitbread et al., 2021). The NHANES data from 2001 to 2006 showed that pregnant females who consumed dairy products had higher urinary iodine concentrations than those who did not report to have consumed dairy within the past 24 h (National Institutes of Health Office of Dietary Supplements, 2023).

Plant-based milk alternatives cannot meet iodine needs in the same way, as these products are not significant sources of iodine. One study comparing the iodine content of 30 different milk alternatives found that, on average, these products contained 3.1 ± 2.5 µg/250 mL of iodine (Ma et al., 2016). This is lower than the iodine content of cow milk. Of course, there are plant-based milk alternatives available on the market that are fortified with certain nutrients to match the nutrient profile of cow milk. However, even these fortified milk alternatives are typically not fortified with iodine because it can alter the flavor profile of the product (Mayo Clinic, 2023). Iodine can have a metallic aftertaste, so many milk-alternative companies do not want to change the flavor of their product to match the iodine content of cow milk.

Dietary guidance on intake of iodine-rich foods in conjunction with the iodization of salt can help to combat low dietary iodine intake, both during pregnancy and in the general population. Dairy foods including milk can help pregnant individuals achieve the recommended iodine intake of 220 to 250 µg/d (Rogan et al., 2014; Alexander et al., 2017; World Health Organization, 2022; National Institutes of Health Office of Dietary Supplements, 2023; American College of Obstetricians and Gynecologists, 2024). For individuals who follow specific dietary patterns (such as various vegetarian dietary patterns) and may not consume dairy foods or seafood rich in iodine, special care should be taken by the pregnant individual's healthcare provider to discuss alternate dietary sources or iodine supplementation to ensure adequate iodine status is achieved. Lactose-free milk and other dairy foods may be an important source of iodine for people with lactose intolerance.

Iodine is an important trace mineral due to its role in supporting metabolism, growth, and brain development (Glinoer, 2001). Iodine is particularly important during pregnancy to support TH production of both mother and baby. Dairy products, including milk, yogurt, and cheese, are good sources of iodine and will provide adequate amounts of this nutrient when consumed in accordance with DGA recommendations. Other than fortified soy products, plant-based milk alternatives are not recommended as dairy food servings by the DGA. Possible ways of consuming more dairy milk throughout the day include making oatmeal using milk, adding milk to smoothies or cereal, or, of course, drinking a glass of milk with meals.
